# Adjusting for Conditional Bias in Process Model Simulations of Hydrological Extremes: An Experiment Using the North Wyke Farm Platform

**DOI:** 10.3389/frai.2020.565859

**Published:** 2020-10-09

**Authors:** Stelian Curceac, Peter M. Atkinson, Alice Milne, Lianhai Wu, Paul Harris

**Affiliations:** ^1^ Rothamsted Research, Department of Sustainable Agriculture Sciences, Devon, United Kingdom; ^2^ Lancaster Environment Centre, Lancaster University, Bailrigg, Lancaster, United Kingdom; ^3^ Geography and Environment, University of Southampton, Highfield, Southampton, United Kingdom; ^4^ State Key Laboratory of Resources and Environmental Information System, Institute of Geographical Sciences and Natural Resources Research, Chinese Academy of Sciences, Beijing, China; ^5^ Rothamsted Research, Department of Sustainable Agriculture Sciences, Harpenden, United Kingdom

**Keywords:** peak flow, conditional extreme model, extreme learning machine, process-based model, hybrid, grassland agriculture

## Abstract

Peak flow events can lead to flooding which can have negative impacts on human life and ecosystem services. Therefore, accurate forecasting of such peak flows is important. Physically-based process models are commonly used to simulate water flow, but they often under-predict peak events (i.e., are conditionally biased), undermining their suitability for use in flood forecasting. In this research, we explored methods to increase the accuracy of peak flow simulations from a process-based model by combining the model’s output with: a) a semi-parametric conditional extreme model and b) an extreme learning machine model. The proposed 3-model hybrid approach was evaluated using fine temporal resolution water flow data from a sub-catchment of the North Wyke Farm Platform, a grassland research station in south-west England, United Kingdom. The hybrid model was assessed objectively against its simpler constituent models using a jackknife evaluation procedure with several error and agreement indices. The proposed hybrid approach was better able to capture the dynamics of the flow process and, thereby, increase prediction accuracy of the peak flow events.

## Introduction

In the United Kingdom, the estimated yearly cost of damages caused by floods is over £1 billion (Collet et al., 2017). Accurate and reliable forecasting of extreme flow events is crucial for planning and implementing measures to mitigate their effects and so protect lives, properties and services. The magnitude and frequency of floods is likely to increase as a result of climate change (Kundzewicz et al., 2007; Bates et al., 2008; Field et al., 2012) and this could push ecosystems beyond the threshold of normal disturbance (Thibault and Brown, 2008). Increased runoff and flooding intensify erosion and result in higher sediment and nutrient losses that can lead to soil degradation and high concentrations of pollutants in water courses (Bouraoui et al., 2004).

Over recent decades, different approaches have been proposed for more accurate modeling and forecasting of peak flows with reduced uncertainty. The two main methods of modeling hydrological variables are physically-based models and statistical models. However, there is an increasing trend toward combining these approaches in hybrid models. One of the most common ways to do this is to post-process statistically an ensemble of forecasts from process-based models (e.g., Cloke and Pappenberger, 2009; Li et al., 2017). Bayesian methods using climate indices (Bradley et al., 2015), stochastic data-driven methods on wavelet decomposed series (Quilty et al., 2019), Bayesian model averaging (Raftery et al., 2005), extended logistic regression (Roulin and Vannitsem, 2011), quantile regression (López López et al., 2014), bias correction (Li et al., 2019) and nearest neighbor resampling for uncertainty estimation (Sikorska et al., 2015) are among the many post-processing techniques described in the literature. Examples of combining a process-based model with more than one statistical or machine learning model can be found in Bogner et al. (2017), Papacharalampous et al. (2019) and Tyralis et al. (2019). The usefulness of combining deterministic and stochastic models (Box and Jenkins, 1976) in real-time flood forecasting was reported by Toth et al. (1999), while the performance of various post-processing techniques according to the level of flow was investigated in Bogner et al. (2016) and Papacharalampous et al. (2019). Hybrid methods for water flow (streamflow) forecasting also include the combination of classical statistical methods with more data-driven, machine-learning methods such as artificial neural networks (ANNs) (Yaseen et al., 2016; Chen et al., 2018; Zhou et al., 2018), discrete wavelet transforms and support vector machines (Kisi and Cimen, 2011), and coupling ANNs with autoregressive techniques (Fathian et al., 2019). The effect of catchment characteristics on the predictive performance of two different statistical models was discussed in Dogulu et al. (2015).

Hydrological process-based models (PBMs) are traditionally used for streamflow modeling and forecasting, where under-prediction of peak flows is a common issue (e.g., Lane et al., 2019; Wijayarathne and Coulibaly, 2020). The PBM performance can suffer from uncertainty due to both random and systematic errors. Both random and systematic errors can arise in the estimated model parameters and measured input variables. However, of particular interest is a type of systematic error (or bias) called conditional bias that depends on flow magnitude. That is, the structure and parameters of the model can generalize the outputs leading to conditional bias, specifically under-prediction of large values and over-prediction of small values; an effect similar in nature to that of having a support that is larger than ideal. Alternatively, data-driven methods may be used, especially when the initial conditions and the parameters of the physical model are difficult to estimate or when the length and/or quality of the data are insufficient for a reliable model calibration.

In this research, we explored combining statistical and machine learning techniques with flow simulations obtained from a PBM to increase the accuracy of forecasting peak flow events. Specifically, we considered the semi-parametric, conditional extreme model (CEM) of Heffernan and Tawn (2004) (a statistical model) and the extreme learning machine (ELM) of Huang et al. (2006) (a machine learning model). The proposed approach is considered a generic solution for enhancing any given hydrological PBM.

The CEM is appropriate for describing the probability that one or multiple variables are extreme and has been applied widely for flood risk analysis (Mendes and Pericchi, 2009; Lamb et al., 2010; Keef et al., 2013; Zheng et al., 2014). A significant property of the CEM is that it is flexible in modeling different dependence structures, such as the dependence of different variables at the same site or the dependence of the same variable at different sites. A key assumption of the application of the CEM is that the extremes of each variable must be independent and, consequently, cannot be used to model peak flow events that have a duration of several consecutive days and, therefore, exhibit temporal dependence. For this reason, the maximum flow during each event was modeled using the CEM while all other peaks were modeled using the ELM (and, thus, a 3-model rather than a 2-model hybrid is proposed).

The ELM model is ANN-based and has been used in various areas of water resources engineering, with a recent focus on water flow (see Yaseen et al., 2019 for an extensive review). In this context, it has been shown to increase accuracy and reduce computational time compared to commonly used benchmark models (Lima et al., 2015) and to other ANN models (Deo and Şahin, 2016).

The resultant 3-model hybrid was evaluated empirically using measured flow data from a sub-catchment of the North Wyke Farm Platform, a grassland research facility in south-west England (Orr et al., 2016). To our knowledge, no study to-date has used the CEM and the ELM to improve the simulation of peak flow events obtained from a PBM, or in which they are combined. The proposed methodology builds on the modeled dependence structure between measured and PBM-simulated peak flow events and uses this relationship to obtain a more accurate representation of these events.

## Methods

This section presents a general description of the CEM (Heffernan and Tawn, 2004) and the ELM (Huang et al., 2006) and explains how they can be applied to peak flow events obtained from a chosen PBM (described in *Choice of Process-Based Model*) in a hybrid context. The flow threshold, above which the simulated and the observed data are considered as possible peaks, is determined based on Generalized Pareto Distribution (GPD) stability plots of the PBM simulated values (Curceac et al., 2020). The performance of the proposed hybrid approach is evaluated using a jackknife procedure and by calculating several error and agreement indices.

### Generalized Pareto Distribution

We characterize peak flow events by fitting the GP distribution to the extreme flow above a certain threshold. The cumulative distribution function (CDF) of the iid excesses over an appropriately high threshold u for the GPD is:

$$G\left(x\right)=\mathrm{Pr}\left(X-u<x|X>u\right)=\left\{ \begin{array}{l} 1-{\left(1+\frac{\xi \left(x-u\right)}{\sigma }\right)}^{-\frac{1}{\xi }},\;\xi \neq 0\\ 1-e^{\left(-\frac{x-u}{\sigma }\right)},\;\;\xi =0 \end{array}\right.$$

where x, for this study, is the peak flow in mm d^−1^, u is the location parameter, σ is the scale parameter and ξ is the shape parameter. The value of the shape parameter defines the type of distribution from the GPD family; that is, ξ=0 refers to the exponential distribution, the distribution has an upper bound of u− σ/ξ when ξ<0 and has no upper limit when ξ≥0.

The first step in modeling the exceedances is to select a threshold over which peaks in flow are considered extreme. The next step is to ensure that the peaks above it are independent (so as to conform with iid) and estimate the scale and shape parameters. The selection of the threshold is a crucial step in GPD extreme value analysis and is basically a trade-off between bias (low threshold-large sample size) and variance (high threshold-small sample size).

The flow threshold in this research was selected based on the simulated flow from the study’s PBM using an automated threshold stability method (Curceac et al., 2020) (*Generalized Pareto Distribution Threshold Selection*) and the same threshold was used for the measured flow data. The GP model was fitted initially independently to the simulated and observed peak flows and the conditional dependence structure between them was estimated using the CEM (*Conditional Extreme Model*).

### Generalized Pareto Distribution Threshold Selection

If the GPD is an appropriate model for the excesses above a threshold u, then for all larger thresholds $u^{*}>u$ it will also be suitable with the shape parameter being relatively constant (Coles, 2001; Scarrott and MacDonald, 2012). That is, it is the approximately linear and horizontal segment on a plot of shape parameter against threshold. This does not apply for the scale parameter $\sigma_{u^{*}}$, which changes with the threshold $\sigma_{u^{*}}=\sigma_{u}+\xi \left(u^{*}-u\right)$. However, the modified scale parameter $\sigma_{1}=\sigma_{u^{*}}-\xi u$ remains relatively constant. Therefore, following Curceac et al. (2020), we fitted a cubic smoothing spline to this plot and calculated the rate of change at each of m consecutive steps. The cubic smoothing spline estimate $\hat{f}$ of a function f in the model $Y_{i}=f\left(x_{i}\right)+\epsilon_{i}$, is defined as the minimizer of $\overset{n}{\underset{i=1}{\sum }}{\left\{Y_{i}-\hat{f}\left(x_{i}\right)\right\}}^{2}+\lambda \int^{\text{​}}{\hat{f}}^{''}{\left(x\right)}^{2}dx\;$, where λ is the smoothing parameter. The minimum change rate locates the part of the plot where the shape and the modified scale parameters reach a plateau.

### Conditional Extreme Model

For a continuous *d-*dimensional vector variable $X=\left(X_{1},\ldots ,\;X_{d}\right)$ with unknown distribution function F(x), the CEM describes the distribution function of X when it is extreme in at least one component. In other words, it describes the conditional distribution of $X_{-i}|X_{i}>u_{X_{i}}$, where $X_{-i}$ is the vector variable X without the component $X_{i}$.

After estimating the marginal distribution of each $X_{i},i=1,\ldots ,d$ (*Generalized Pareto Distribution*), and before estimating the extremal dependence, the variables are transformed so that they follow the same distribution. This process is called marginal standardization and is used to distinguish the marginal behavior from the dependence structure (Drees and Janßen, 2017). The data can be transformed to either Gumbel margins to describe the positive dependence or to a Laplace marginal distribution which, due to its exponential tail and symmetry, captures both positive and negative dependence (Keef et al., 2013). The initial vector variable X is, therefore, transformed as:

$$f\left(x\right)=\left\{ \begin{array}{l} \log\left\{2F_{X_{i}}\left(X_{i}\right)\right\},\;\;X_{i}<F_{X_{i}}^{-1}\left(0.5\right)\\ -\log\left\{2\left[1-2F_{X_{i}}\left(X_{i}\right)\right]\right\},\;\;X_{i}\geq F_{X_{i}}^{-1}\left(0.5\right) \end{array}\right.$$

where $F_{X_{i}}^{-1}$ is the inverse cumulative distribution function of $X_{i}$. The resulting vector variable $Y=\left(Y_{1},\ldots ,Y_{d}\right)$, therefore, has Laplace margins with:

$$\mathrm{Pr}\left(Y_{i}\leq y\right)=F_{Y_{i}}\left(y\right)=\left\{ \begin{array}{l} \frac{1}{2}\exp\left(y\right),\;\;y<0\\ 1-\frac{1}{2}\exp\left(-y\right),\;\;y\geq 0 \end{array}\right.$$

The dependence model considers the asymptotics of the conditional distribution $\text{Pr}\left(Y_{-i}\leq y_{-i}|Y_{i}=y_{i}\right)$, where for $y_{i}\to \infty$, the increase of $y_{-i}$ must result in non-degenerate margins. For this, assume the normalizing functions ${\text{a}}_{i}\left(y_{i}\right)$ and $b_{i}\left(y_{i}\right)$, that have the same dimension as $Y_{-i}$ and for which:

$$\lim_{y_{i}\to \infty }\left[\mathrm{Pr}\left\{\frac{Y_{-i}-a_{i}\left(y_{i}\right)}{b_{i}\left(y_{i}\right)}\leq z_{i}|Y_{i}=y_{i}\right\}\;\right]=G_{i}\left(z_{i}\right)$$

where the limit distribution $G_{i}$ has non-degenerate marginals $G_{j|i}$ for all j≠i. Therefore, the random variable $Z_{|i}=\frac{Y_{-i}-{\text{a}}_{|i}\left(y_{i}\right)}{b_{|i}\left(y_{i}\right)}$ is independent of $Y_{i}>u_{Y_{i}}$ and has distribution function $G_{|i}$. The location $a_{|i}\left(y_{i}\right)$ and scale $b_{|i}\left(y_{i}\right)$ functions are given by $a_{i}\left(y_{i}\right)=\alpha_{|i}y_{i}$ and $b_{|i}\left(y_{i}\right)={y_{i}}^{\beta_{i}}$ where the vector constants $\alpha_{|i}$ and $\beta_{|i}$ take values of ${\text{α}}_{j|i}\in \left[-1,1\right]$ and $\beta_{j|i}\in \left(-\infty ,1\right)$, respectively, for all j≠i. Finally, the dependence structure is described by the multivariate semi-parametric regression model:

$$Y_{-i}=\alpha_{|i}y_{i}+{y_{i}}^{\beta_{|i}}Z_{|i}\text{ for }Y_{i}=y_{i}>u_{Y_{i}},\;\;i=1,\ldots ,d.$$

The above equation expresses the behavior of the vector variable Y, excluding the element of $Y_{i}$ when it takes a large value. The dependence between the variables $Y_{i}$ and $Y_{j}$ is explained by the constant ${\text{α}}_{ji}$. Positive values indicate a positive relationship. The constant $\beta_{ji}$ incorporates the changes in the variability of $Y_{j}$ as $Y_{i}$ increases. Details on estimating the dependence parameters are given in Heffernan and Tawn (2004) and Keef et al. (2013).

To obtain randomly generated samples of $X|X_{i}>u_{X_{i}}$, we adopted the following procedure. Initially, samples of $Y_{i}$ from the Laplace distribution are simulated conditional on it exceeding its cumulative probability corresponding to $F_{X_{i}}\left(u_{X_{i}}\right)$. Similarly, samples of random observations of $Z_{i}$ are drawn from its estimated distribution ${\hat{G}}_{i}$. Then, using the semi-parametric model, we obtain $Y_{-i}={\hat{\text{α}}}_{i}y_{i}+{y_{i}}^{{\hat{\beta }}_{i}}Z_{i}$ and transform the vector $Y=\left(Y_{-i},Y_{i}\right)$ to the originally distributed $X=\left(X_{-i},X_{i}\right)$ by the inverse transformation.

### Extreme Learning Machine

The ELM is a data-driven method developed by Huang et al. (2006) that has been used effectively for streamflow forecasting (e.g., Deo and Şahin, 2016; Yaseen et al., 2016). Compared to other common ANN techniques, it has the advantages of fast learning speed and is characterized by improved performance in terms of commonly encountered problems, such as over-fitting and the effect of local minima. The model has a three-layer structure with one input, one hidden and a single output layer and can be expressed mathematically as:

$$\overset{\Lambda }{\underset{i=1}{\sum }}B_{i}h_{i}\left(m_{i}\cdot x_{t}+n_{i}\right)=z_{t}$$

where Λ is the total number of nodes, B are the estimated weights between the nodes of the hidden and output layers, and h(m,n,x) is the activation function with weights $m_{i}\in {ℜ}^{d}$, biases $n_{i}\in ℜ$ and the explanatory variable of the training dataset $x_{t}\in {ℜ}^{d}$. Here, i and d denote the index of a specific hidden neuron (HN) and the number of input neurons, respectively, and Z is the model output.

Initially, the ELM model selects the input weights and hidden layer biases at random, and then calculates the output weights using a least squares method instead of adjusting them iteratively (see Chen et al., 2018 for details). Once the output weights $\hat{B}$ have been estimated, forecasts are obtained by substituting the training dataset $x_{t}$ with the testing one. The number of HNs in the hidden layer and the activation function are the only parameters that need to be pre-defined. The optimal number of HNs is a trade-off between generalization ability and network complexity. A highly complex model with too many HNs can lead to over-fitting, whereas a decreased number of HNs can result in a model that is too simple to capture non-linear relationships. The optimal number of HNs is problem-dependent and is frequently determined empirically (Huang et al., 2006; Sun et al., 2008). In this research, the number of HNs was increased iteratively from 1 to 100 and the network structure that provided the smallest RMSE of the training procedure was selected.

### Application and Evaluation

A jackknife evaluation procedure (Miller, 1964; Shao and Tu, 1995) was applied to assess the performance of the proposed hybrid approach. It is a leave-one-out resampling technique without random replacement where one observation or a fixed subset of the dataset is omitted iteratively. The main strengths of the jackknife method are that model accuracy is independent of the calibration data and the loss in the sample data information is minimal (McCuen, 2005).

As stated previously, peak events are defined as flow above a certain threshold of the PBM simulated data. At each iteration, one peak flow event (measured and simulated) was left out of the dataset. This event constitutes the testing dataset and the rest of the data the training dataset, and the CEM and the ELM were fitted to the latter. The dependence behavior of measured peaks conditional on the PBM simulated, above a certain threshold, was configured by the CEM. From the fitted CEM, 50,000 stochastic simulations were obtained for both the observed $X_{j}$ (pseudo-observations) and the PBM simulated $X_{i}$ variables (pseudo-PBM simulated). From the total set of random simulations of the conditioning variable $X_{i}$, the ones with the smallest difference (≤0.1) from the maximum PBM simulated peak of the testing sample, which was left out of the training dataset, were considered. As CEM provides pairs of simulated data according to their dependence structure, the corresponding random simulations of $X_{j}$ (pseudo-observations) were then obtained. By calculating their median value, a forecast of the maximum flow during an event was obtained and compared to the maximum measured and PBM simulated peak excess of the testing dataset.

The ELM model was trained using PBM simulated data as inputs and measured data as outputs of the training dataset. Based on the trained ELM model, flow forecasts were then obtained using the PBM simulated flow of the testing sample as explanatory variable, except for the maximum. Consequently, peaks smaller than the cluster maxima were forecasted by the ELM and the CEM was used only to forecast maximum flows. The application of the ELM model alone on all the peaks was also performed in experimentation and its performance compared to the CEM for the maximum flows. At the next iteration, a different peak flow event was omitted from the training dataset for testing purposes and the same process was repeated for all peaks.

**FIGURE 1:**
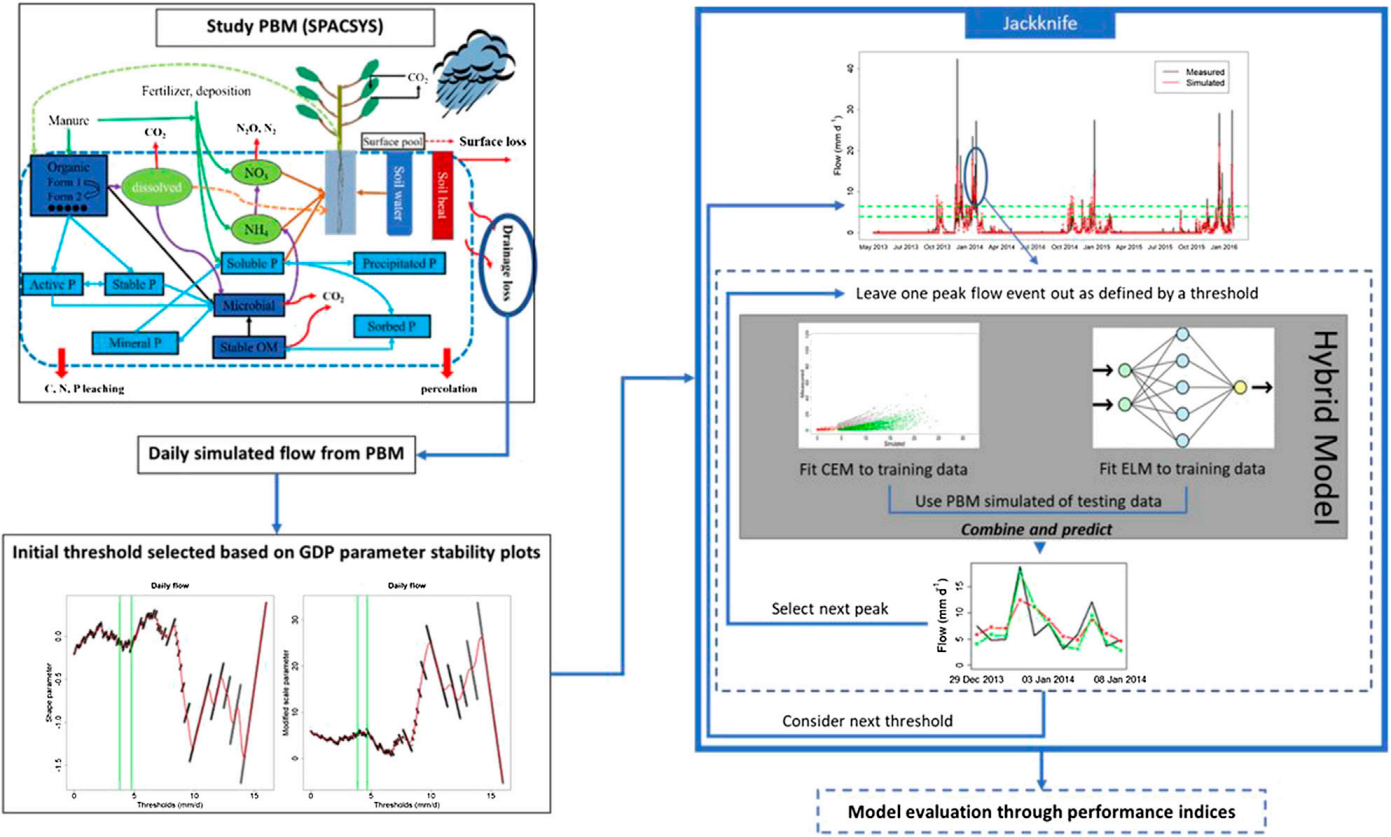
Schematic of the proposed methodology.

This procedure was performed initially for peaks above the threshold that corresponds to the start of the region of stability of shape and modified scale parameters. However, in order to investigate the effect of threshold selection on the proposed methodology, the above-mentioned procedure was repeated for different thresholds. The considered thresholds were set as a range from the minimum that resulted from the application of threshold stability method, up to the 95th quantile of the PBM simulated flow. Higher thresholds resulted in data scarcity that did not allow the models to be fitted satisfactorily. All the above-mentioned steps are presented diagramatically in Figure 1.

To assess the accuracy of the peak flow forecasts for each threshold, a set of indices was calculated. More specifically, the mean absolute error (MAE), the normalized root mean square error (NRMSE), the percentage BIAS (PBIAS), the Nash-Sutcliffe efficiency (NSE), the index of agreement (*d*) and the Kling-Gupta Efficiency (KGE) were computed using the following equations:

$$\text{MAE}=\frac{1}{N}\overset{N}{\underset{i=1}{\sum }}\left|{\hat{z}}_{i}-z_{i}\right|$$

$$\text{NRMSE}=100\frac{\sqrt{\frac{1}{N}\sum_{i=1}^{N}{\left({\hat{z}}_{i}-z_{i}\right)}^{2}}}{z_{\max}-z_{\min}}$$

$$\text{PBIAS}=100\frac{\sum_{i=1}^{N}\left({\hat{z}}_{i}-z_{i}\right)}{\sum_{i=1}^{N}z_{i}}$$

$$\text{NSE}=1-\frac{\sum_{i=1}^{N}{\left({\hat{z}}_{i}-z_{i}\right)}^{2}}{\sum_{i=1}^{N}{\left(z_{i}-{\bar{z}}_{i}\right)}^{2}}$$

$$d=1-\frac{\sum_{i=1}^{N}{\left({\hat{z}}_{i}-z_{i}\right)}^{2}}{\sum_{i=1}^{N}{\left(\left|{\hat{z}}_{i}-{\bar{z}}_{i}\right|+\left|z_{i}-{\bar{z}}_{i}\right|\right)}^{2}}$$

$$\text{KGE}=1-\sqrt{{\left(r-1\right)}^{2}+{\left(\frac{\sigma_{\hat{z}}}{\sigma_{z}}-1\right)}^{2}+{\left(\frac{\bar{\hat{z}}}{\bar{z}}-1\right)}^{2}}$$

where ${\hat{z}}_{i}$ are the simulated (or predicted) values, $z_{i}$ are the measurements (or observed values), ${\bar{z}}_{i}$ is the mean of the measured values, r is the Pearson product-moment correlation coefficient (between ${\hat{z}}_{i}$ and $z_{i}$) and σ is the standard deviation. The optimal value of the error indices (MAE, NRMSE, and PBIAS) is zero and the smaller are the values, the more accurate are the simulations. NSE (Nash and Sutcliffe, 1970) takes values from −∞ to 1, where one corresponds to a perfect match between simulated and measured values, zero indicates that model simulations are as accurate as the mean of the measured values and a negative value indicates that the mean of the measured values is a more accurate predictor than the model. The index of agreement, d is defined in the range of zero to one, where again one represents the perfect model and zero no agreement at all. KGE incorporates r, the ratio between the means of the measurements and the simulations, and the variability ratio. KGE takes the same value range as NSE.

## Study Site and Data

### Study Site

The flow discharge data used in this research were measured at the North Wyke Farm Platform (NWFP). The NWFP is a farm-scale experiment established in 2010 in the southwest of England (50°46′10″N, 3°54′05″W) to support research into sustainable grassland livestock systems (Orr et al., 2016). The platform comprises three independent small farms, each 21 ha in size. Each farm is divided into five sub-catchments, with some sub-catchments consisting of more than one field. The platform monitors routinely water run-off and water chemistry in each of the 15 sub-catchments, together with other primary data collections (e.g., greenhouse gas emissions) so that each farming system can be evaluated according to its level of sustainability (Takahashi et al., 2018). For the period 1985–2015, the average annual temperature at North Wyke ranges from 6.8 to 13.4°C and the average annual rainfall is 1,033 mm. The platform has an altitude range of 120–180 m above sea level. Soil texture consists of a slightly stony clay loam topsoil (about 36% clay) above a mottled stony clay (about 60% clay). The subsoil is impermeable to water and during rain events most of the excess water moves by surface and sub-surface lateral flow toward the drainage system described below.

**FIGURE 2:**
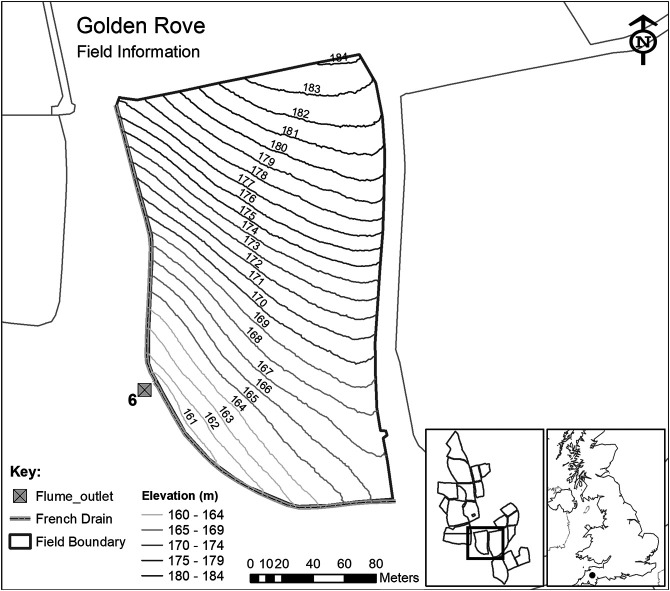
Details of the sub-catchment selected for this research from the total of 15 sub-catchments within the North Wyke Farm Platform.

Each of the 15 sub-catchments (inset in Figure 2) are hydrologically isolated through a combination of topography and a network of French drains (800-mm deep trenches) which ensure that the total runoff is channeled to instrumented flumes, measuring water discharge and its chemistry with a 15 min temporal frequency since October 2012. The runoff from each sub-catchment is measured through a combination of primary and secondary flow devices. The primary devices are H-type flumes (TRACOM Inc,, Georgia, USA) with capacity designed for a 1-in-50-year storm event (in respect of data preceding 2010). The specific design of the H-type flume facilitates the accurate measurement of both low and high flows and is relatively self-cleaning since it allows the ready passage of sediment and particulate matter. A secondary flow measurement device (OTT hydromet, Loveland, CO, USA) is used to measure the water height within the flume and convert it to discharge rate using flume-specific formulas which depend on water height. The flow is generated only from rainfall as the fields are not irrigated. Each sub-catchment also monitors precipitation and soil moisture every 15 min.

Platform data acquired from October 2011 to July 2013, represent a baseline period where all farm fields were categorized as permanent pasture and received identical rates of inorganic fertilizers and farmyard manure. From July 2013 to July 2015, two of the three farms entered a transition phase and were ploughed and reseeded progressively with different types of pasture; specifically, a mixture of white clover and high sugar perennial ryegrass, and sugar perennial ryegrass only. Thus, two farms entered fully a post-baseline period in July 2015.

For this research, we used flow discharge (from April 2013 to February 2016) measured at sub-catchment six of the permanent pasture farm (Figure 2), which consists of a single field (Golden Rove). This field was chosen because, as part of the permanent pasture farm, it would not have been ploughed and reseeded during the period of study (which would affect various processes, such as runoff).

### Choice of Process-Based Model

For this research, we used the “SPACSYS” model to simulate the flow discharge for sub-catchment six of the NWFP over the period of interest. The SPACSYS model is a process-based, field-scale model which simulates key agricultural processes such as plant growth and development, soil Carbon and Nitrogen (N) cycling, water dynamics and heat transformation (Wu et al., 2007) (see Figure 1). The main processes concerning plant growth are assimilation, respiration, water and N uptake, partitioning of photosynthate and N,N-fixation for legume plants and root growth. The Richards equation for water potential is used in SPACSYS to simulate water redistribution in a soil profile. Site-specific input data for the simulations include daily weather variables from the North Wyke site, soil properties, field and grass management (e.g., fertilizer application dates and composition, reseeding, grazing and cutting dates), and initialization of the state variables (standing biomass and root distribution, soil water and temperature distribution). Previous simulations of water runoff, soil moisture and other agricultural processes for sub-catchment six of the NWFP using SPACSYS can be found in Liu et al. (2018), where a detailed explanation on the SPACSYS calibration is given.

## Results

### Comparison of Measured Flow Data With Process-Based Model Simulations

**FIGURE 3:**
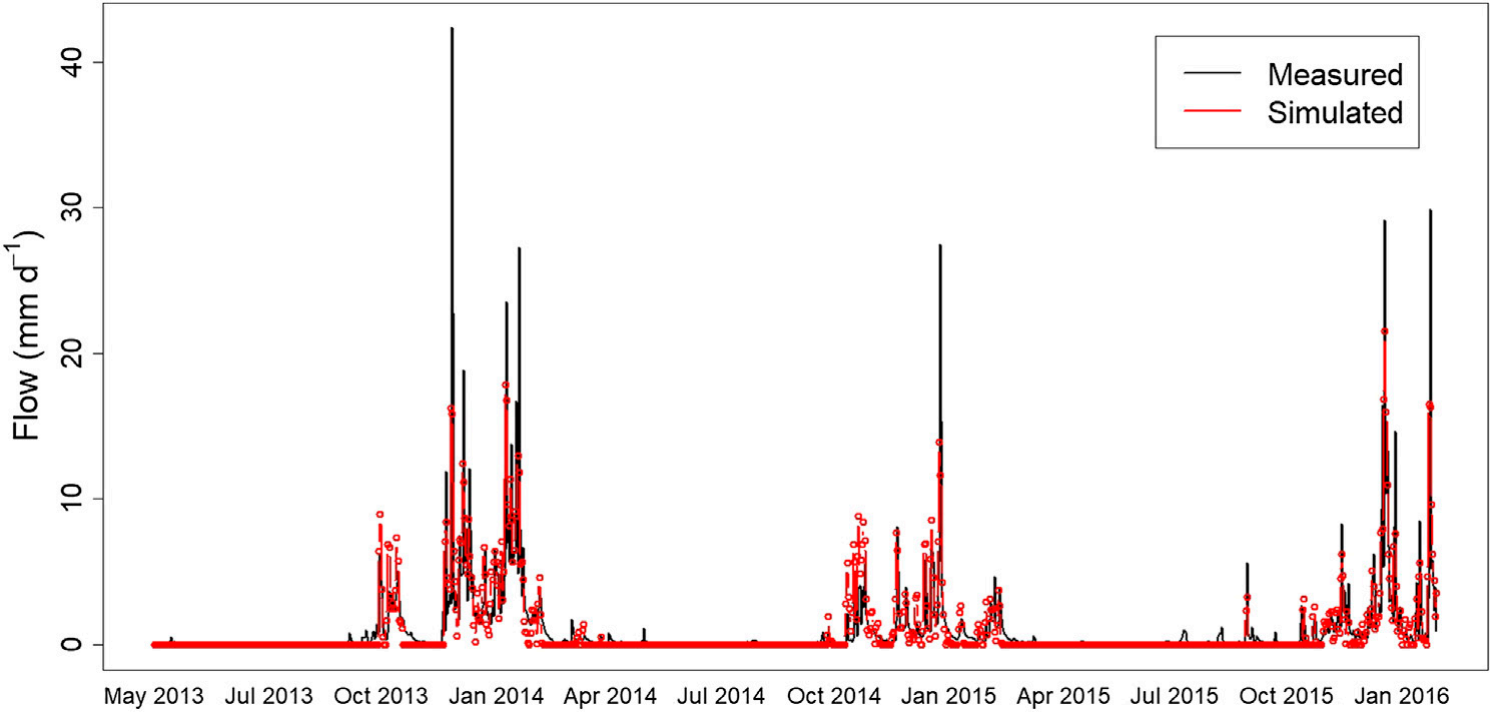
Time-series of measurements and PBM simulation of flow (mm d^−1^) at the study site from May 2013 to February 2016.

**FIGURE 4:**
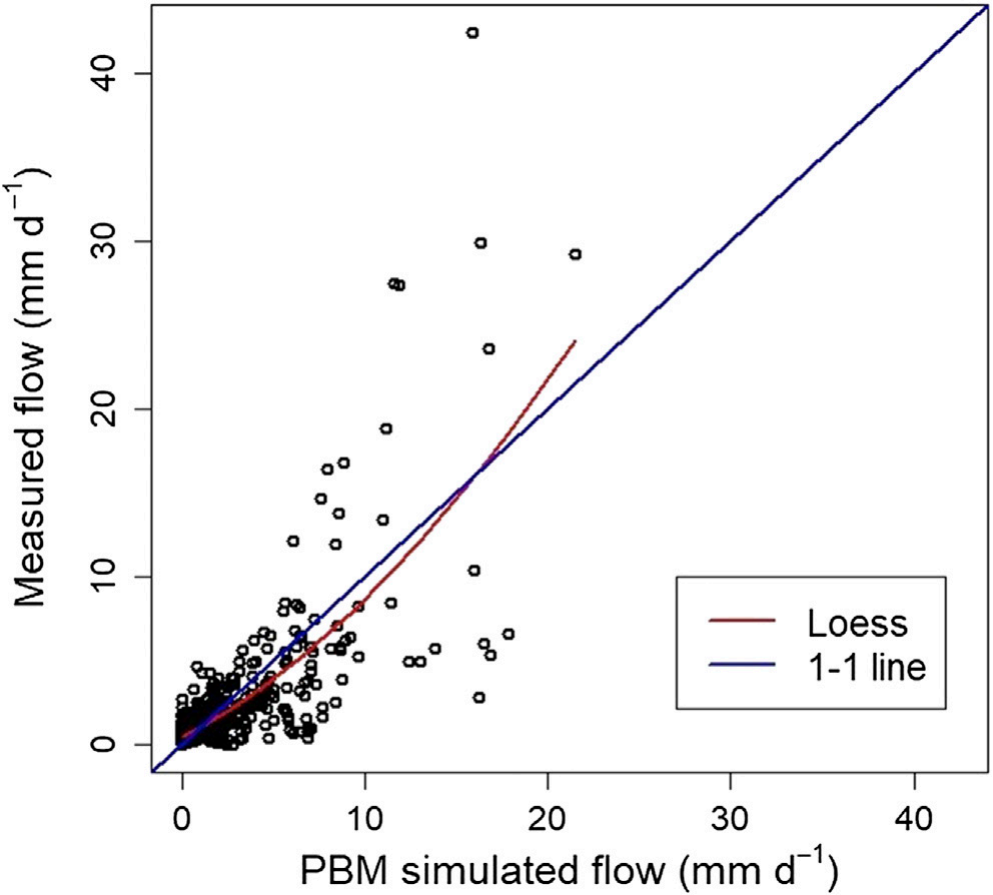
Scatterplot of measurements of flow (mm d^−1^) against PBM simulated flow at the study site. The scatterplot is shown with the ideal 1:1 line and a Loess smoother fit.

The plotted time-series of measured and PBM simulated flow (Figure 3), shows that the simulation appears to capture well the general behavior of the process at low flows. However, it tends to under-predict the high flows and over-predict the medium ones. This is confirmed by the corresponding scatterplot (Figure 4) where many values in the range 5–10 mm d^−1^ are below the 1-to-1 line and, thus, the simulated flow is greater than that measured. A non-linear locally weighted regression fit (i.e., a Loess smoother, see Cleveland, 1979), to the measured and simulated data is also given to help illustrate this behavior.

### Threshold Selection

**FIGURE 5:**
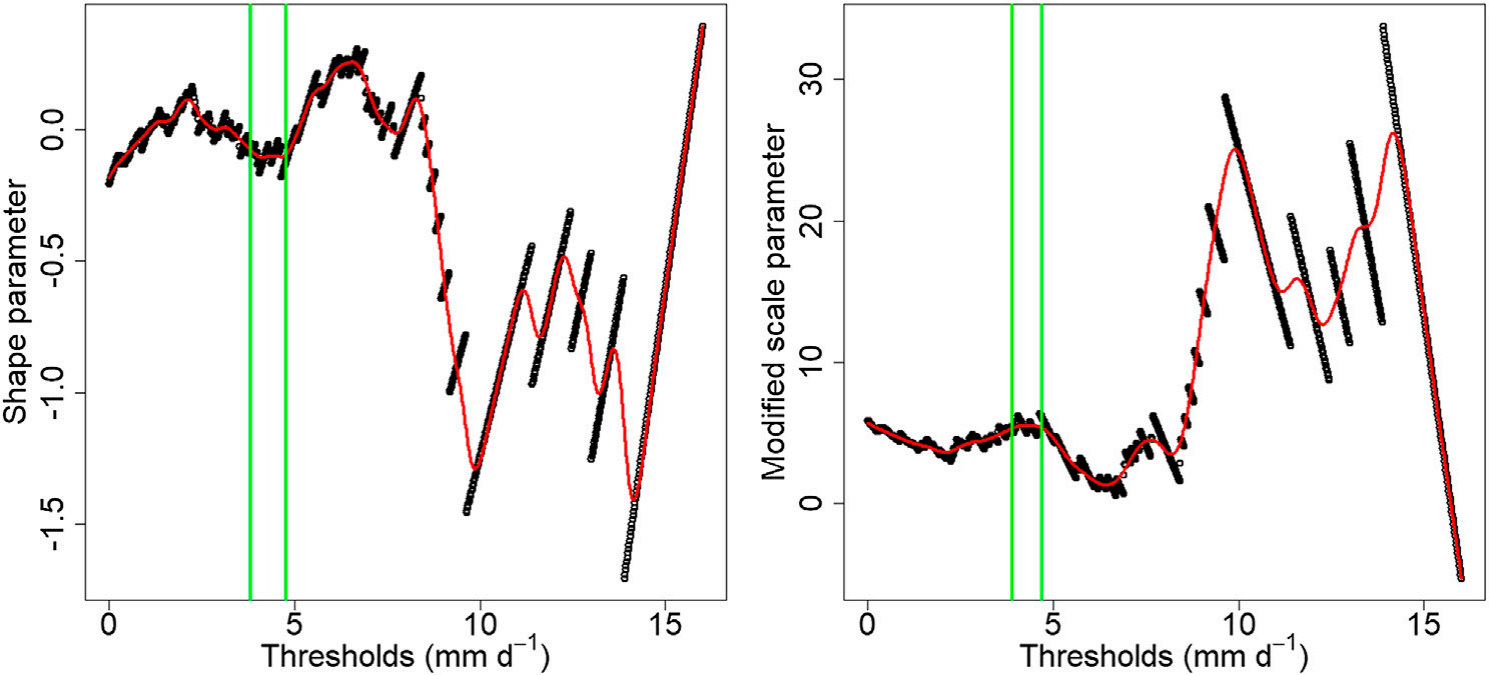
Shape and modified scale parameters for different threshold candidates applied to the PBM simulated daily flow. The red lines are the fitted splines and the green vertical lines specify the selected region of stability.

The shape and modified scale parameters estimated using the method of Curceac et al. (2020) indicated very similar threshold choices, in regions where the parameters remained relatively stable for increasing threshold candidates (Figure 5). The minimum threshold according to the shape parameter is 3.96 mm d^−1^ and according to the modified scale parameter, 3.88 mm d^−1^. These thresholds were estimated based on the PBM simulated flow (as described above), and the same thresholds were used for the observed peaks. Diagnostics, such as QQ plots of the empirical and modeled distributions (not presented), indicated that the GPD provides a good fit to the excesses and can model satisfactorily the peaks above the threshold of 3.88 mm d^−1^, which was eventually selected. The range of thresholds above which the models where applied, was set from 3.88 up to 6.41 mm d^−1^, with the maximum corresponding to the 95th quantile of the PBM simulated flow.

### Conditional Extreme Model Fit

**FIGURE 6:**
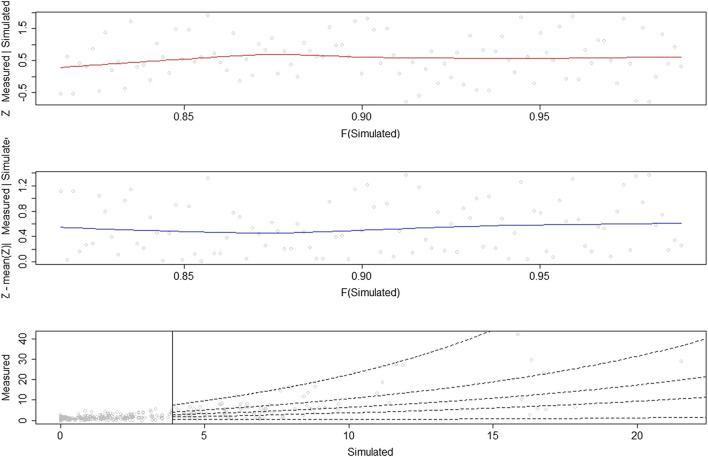
Diagnostic plots for the fitted extreme dependence model (CEM): **(top)** scatterplot of the residuals Z against the conditioning PBM simulated data with a Loess curve (in red) for the local mean values; **(middle)** absolute of the normalized residuals Z against the conditioning PBM simulated data with a Loess curve (in blue); **(bottom)** scatterplot of measured vs. PBM simulated data, with the fitted quantiles of the distribution of measured data conditional on PBM simulated data (dashed lines).

**FIGURE 7:**
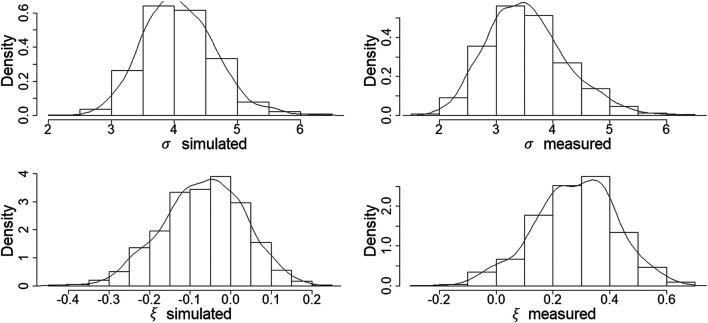
Bootstrap-estimated distributions of the scale and shape parameters (**top and bottom histograms,** respectively) for the conditioning (PBM simulated) and dependent (measured data) variables (**left and right histograms,** respectively).

**FIGURE 8:**
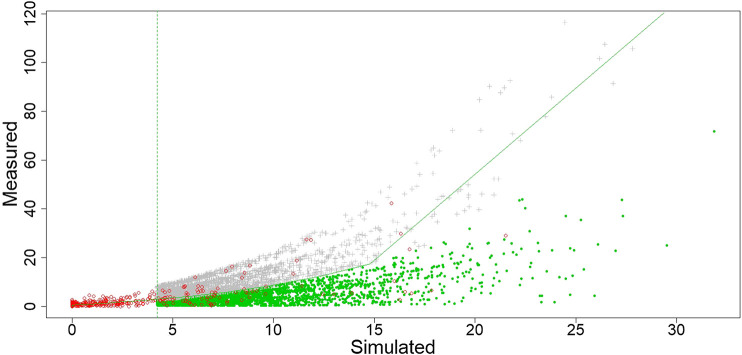
Scatterplot of measured vs. PBM simulated flow (red circles) together with CEM simulated data (gray crosses and green circles) plotted above the threshold for prediction (green, dashed vertical line). The fitted curve (green solid line) joins equal quantiles of the marginal distributions and is used only for reference.

The diagnostics of the extreme dependence model (CEM) show a satisfactory fit (Figure 6). As stated in *Conditional Extreme Model*, one of the main assumptions of the model is that the residuals Z are independent of the conditioning variable (in this case, the PBM simulations). The pattern of both the initial and absolute values of the normalized residuals conforms approximately to a uniform distribution with no distinct pattern in the location or scatter of these residuals with the conditioning PBM simulations. The slight trend in the residuals Z for the lowest peaks of the conditioning variable might indicate that a higher threshold should be considered. The fitted quantiles of the conditional distribution of the dependent variable (measured data) conditional on the PBM simulated data (Figure 6, bottom) shows a good agreement between the data and the fitted quantiles, which capture the whole range of the scatter. Histograms of the scale and shape parameters (Figure 7) show that the measured and PBM simulated peaks have similar scale characteristics. However, the distribution of the measured peaks has a considerably heavier tail $\left(\xi_{obs}>\xi_{sims}\right)$. The CEM simulated values of the dependent variable (measured data) along with the values of the conditional variable (PBM simulated data) (Figure 8) were obtained using the CEM with estimated dependence parameters of α = 0.44 and β = 0.59. These parameters confirm that there is a positive dependence between the measured and the PBM simulated data, and that the measured data increase in variability as the values of the PBM simulations increase.

### Hybrid Model via Conditional Extreme Model-Extreme Learning Machine Adjustments of Process-Based Model Simulated Data

**FIGURE A1:**
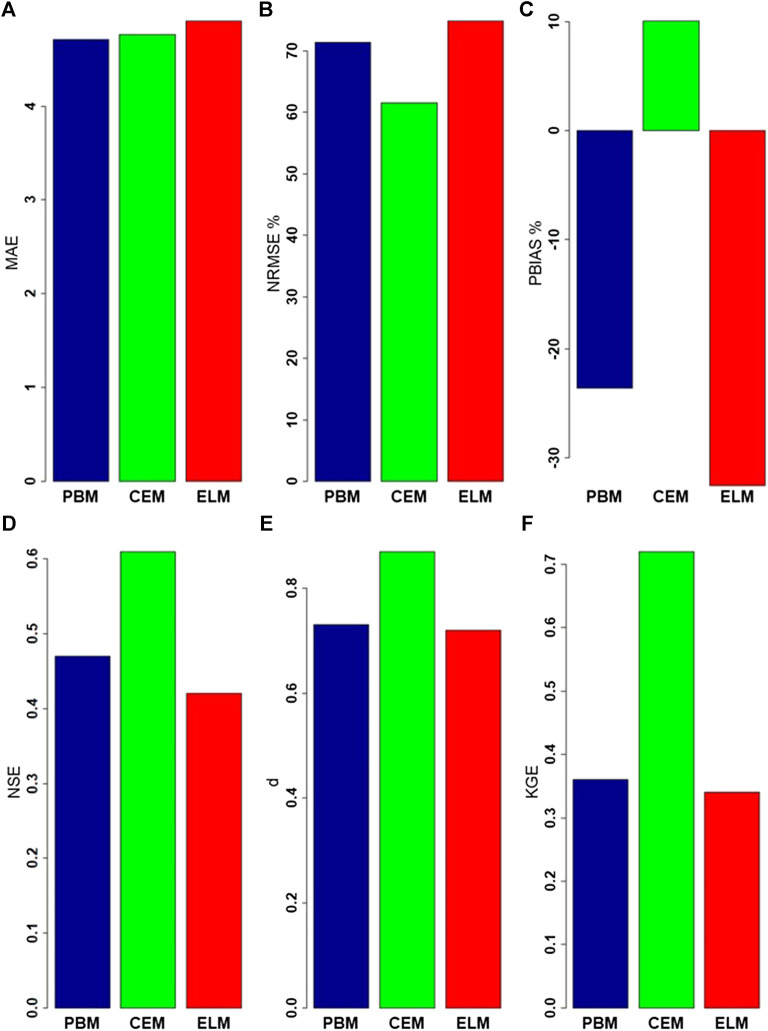
Error and agreement indices of the process-based model, conditional extreme model, and extreme learning machine simulated maximum peaks compared to observed data. **(A)** MAE, **(B)** NRMSE, **(C)** PBIAS, **(D)** NSE, **(E)** *d*, **(F)** KGE.

To recap, this research applies the CEM for the maximum peaks, while the ELM model is used for the smaller peaks during a peak flow event as the ELM alone did not increase the accuracy of the maximum peaks (over that found with the PBM alone). For reference, error and agreement performance indices are given in **Appendix** (Figure A1) for the three constituent models of the study hybrid (i.e., for PBM only, CEM only, and ELM only), for predicting the maximum peaks.

**FIGURE 9:**
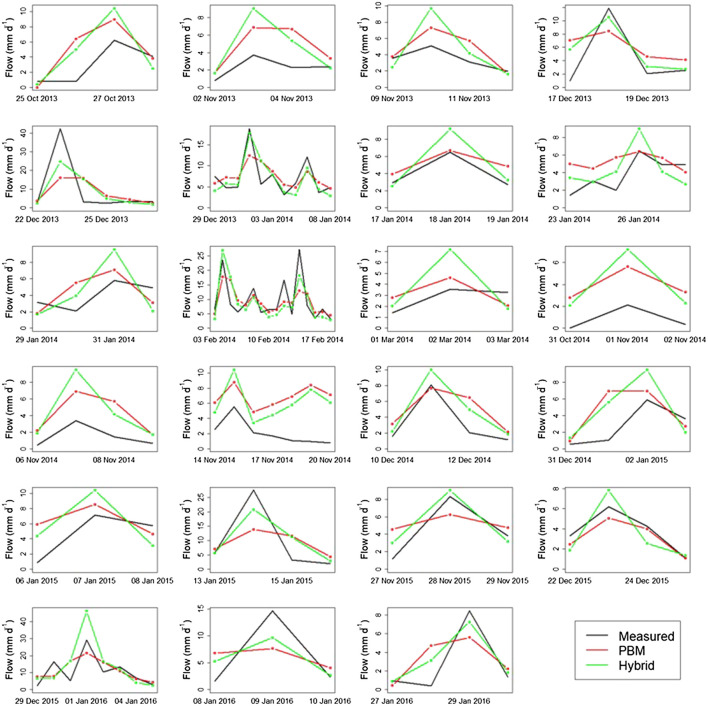
Time-series plots of measured, PBM-predicted and hybrid model-predicted flow for all considered peak flow events for which the PBM simulated data >3.88 mm d^−1^, following the threshold selection analysis of *Discussion*.

The resultant hybrid simulations (or adjusted PBM simulations) for peak flow events above the minimum threshold of 3.88 mm d^−1^ are presented in Figure 9 together with the PBM simulated data and the measured data. The PBM most commonly under-predicts the largest peaks and over-predicts the ones preceding and following it. Use of the CEM captures the cluster maxima more accurately, which naturally depends on the value of the PBM simulation. In cases where the PBM over-predicts the maximum peak, the CEM leads to an even greater error. The ELM model addresses the fact that the PBM tends to over-predict the smaller peaks and, thus, provides hybrid forecasts of these peaks that are smaller and closer to the measured ones. The characteristics of the elements of the proposed methodology, in combination, results in improved characterization of the peak flow events, that tend to rise and fall more steeply (and realistically) than is found with the PBM simulations. Key exceptions arise for cases where the PBM over-predicts the whole event, as the hybrid compounds this over-prediction.

**FIGURE 10:**
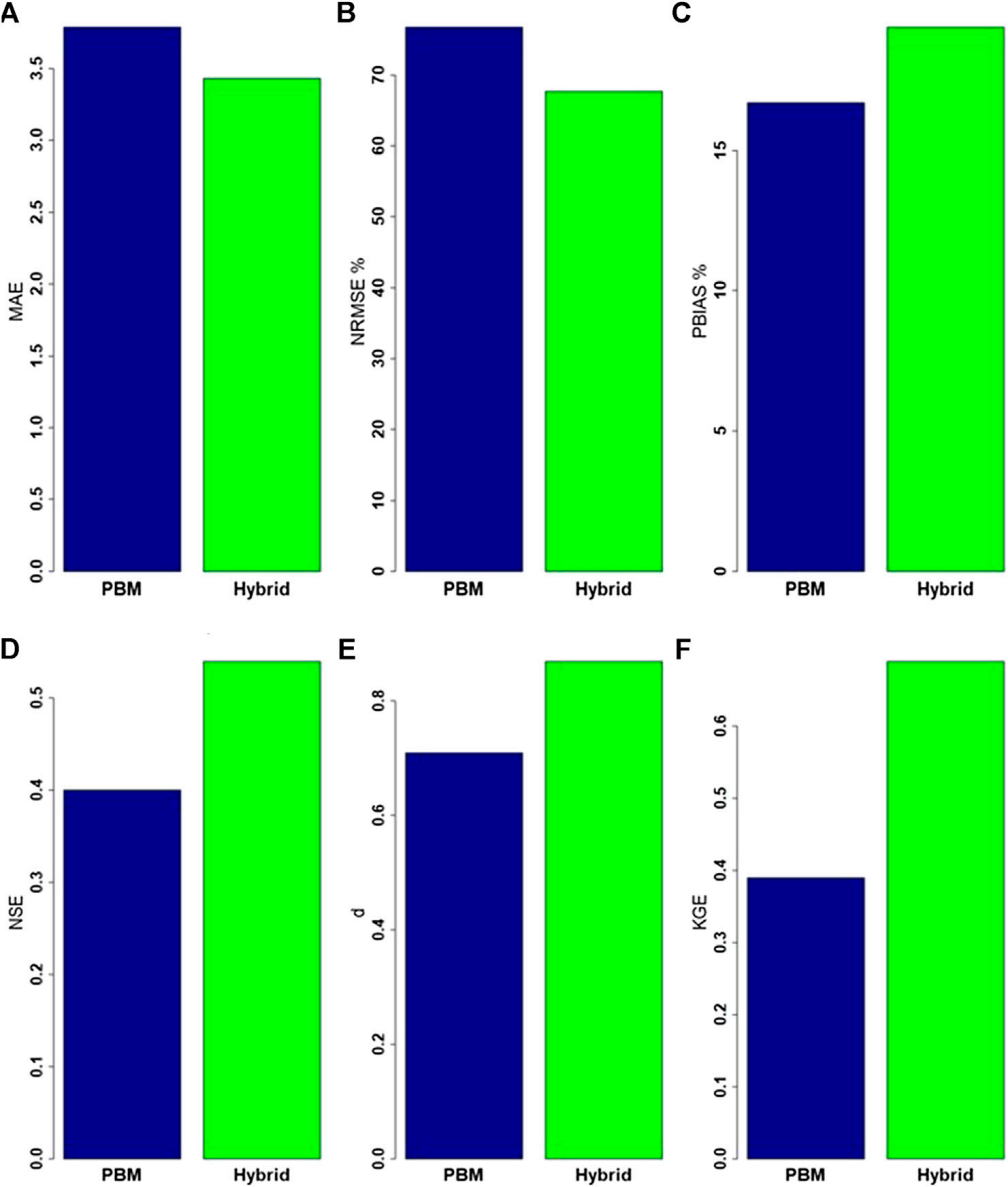
Error and agreement indices of the PBM and hybrid simulated data compared to observed data: **(A)** mean absolute error (MAE), **(B)** the normalized root mean square error (NRMSE), **(C)** the percentage BIAS (PBIAS), **(D)** the Nash-Sutcliffe efficiency (NSE), **(E)** the index of agreement (*d*) and **(F)** the Kling-Gupta Efficiency (KGE)

Error and agreement indices (Figure 10) provide an overall assessment of the proposed hybrid methodology for the same peak flow events (of Figure 9), but specifically just for instances of PBM simulations >3.88 mm d^−1^. In general, the proposed hybrid approach is more accurate, as it results in smaller error indices and larger agreement indices than produced using the PBM alone, except for PBIAS, despite reductions in the other two error indices (MAE and NRMSE). Clearly, PBIAS is more reflective of how the hybrid can sometimes compound over-prediction. The greatest relative improvement was found in the KGE index, although both NSE and d also indicated improved agreement between observed and hybrid simulated values.

**FIGURE 11:**
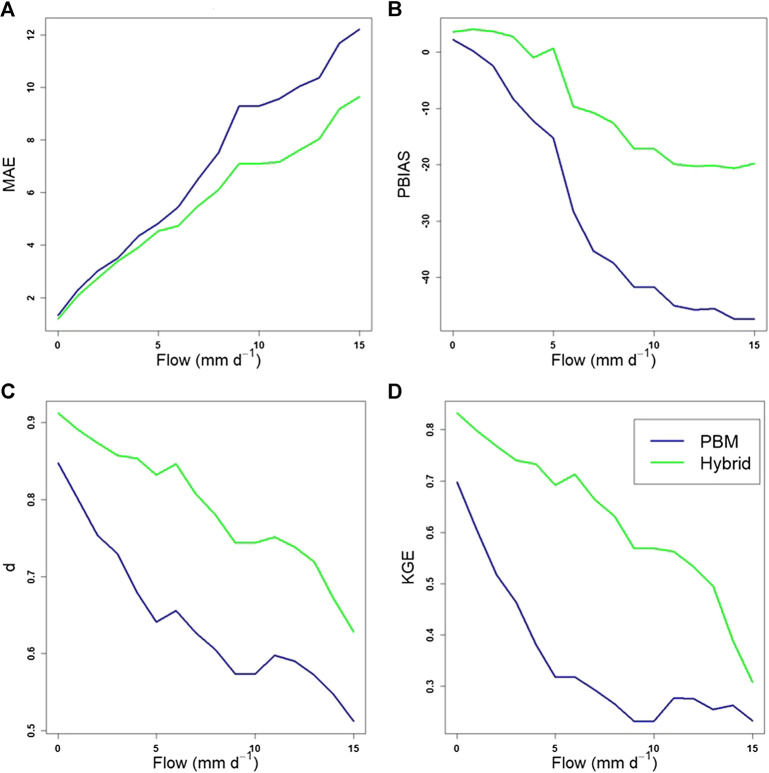
Error and agreement indices of the PBM and hybrid simulated data for increasing observed flow values. **(A)** MAE, **(B)** PBIAS, **(C)** d, **(D)** KGE.

All of the results discussed above relate only to instances of PBM simulated flow values above the threshold of 3.88 mm d^−1^, where the measured and hybrid simulated values directly correspond to. We compare now between *all* the measured water flow data, the PBM and hybrid simulations when above the selected threshold. The resultant plots of error (MAE and PBIAS only) and agreement (d and KGE only) indices against the magnitude of observed flow are given in Figure 11. The MAE is very small for both the PBM and the hybrid when comparing simulated flow with *all* the observed flow above the threshold. Increasing the observed flow threshold above which data are compared with the simulated data, results in a slower increase (with flow magnitude) in the MAE for the hybrid than for the PBM outputs. The hybrid approach also results in a significant decrease of the negative PBIAS with increasing peak flow, relative to the PBM. The agreement indices (d and KGE) similarly confirm this improvement found for the hybrid simulations over the PBM simulations.

**FIGURE 12:**
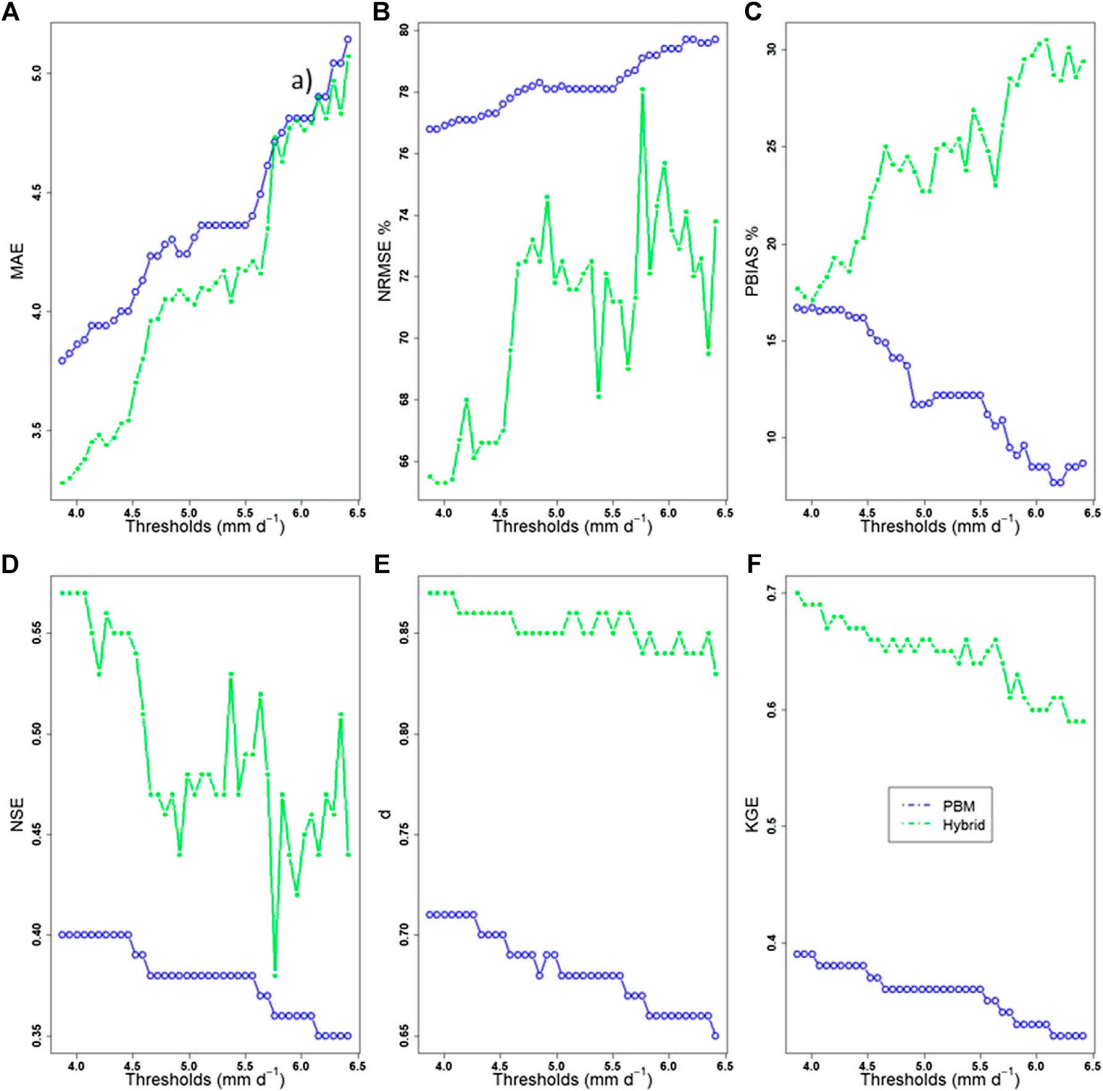
Error: **(A)** MAE, **(B)** NRMSE, and **(C)** PBIAS indices (top three plots) and agreement: **(D)** NSE, **(E)** d,and **(F)** KGE indices (bottom three plots) of the PBM and hybrid simulated data for a range of thresholds (3.88–6.5 mm d^−1^).

All of the results discussed above refer to peak events above the threshold of 3.88 mm d^−1^, as selected based on the GPD parameter stability plots (Figure 5). As a final step in the analysis, it is prudent to assess how threshold selection has an effect on the performance of the proposed methodology. Thresholds were set to range from 3.88 mm d^−1^ up to the 95th quantile of the PBM simulated flow (6.5 mm d^−1^). According to the calculated MAE indices, the hybrid model has a performance similar to the PBM when considering peak events above the threshold of 5.8 mm d^−1^ (Figure 12 **)**. This is not confirmed by the NRMSE which, however, shows a steep increase for the same threshold. PBIAS shows an overall increasing trend with some fluctuations in between. The agreement indices (Figure 12) seem to be less sensitive to the threshold, although NSE shows an abrupt decrease when flow is higher than 5.8 mm d^−1^. All the indices have the common characteristic of the consistent trend (increasing for error, decreasing for agreement) as the threshold increases, which could be attributed to the smaller samples of the data used for testing, in which the highest flow values dominate.

## Discussion

The main motivation for developing the proposed hybrid approach was to forecast more accurately the peak flows that are typically under-predicted using PBMs due to model over-generalization or smoothing. The analysis in this research was based on simulations obtained from the SPACSYS model. SPACSYS has characteristics that can be considered as representative of the vast majority of PBMs used for flow simulations and the hybrid approach presented is entirely general. However, the PBM also exhibited other problems, such as over-predicting small and moderate flow values. This second problem arises because the model (as for most PBMs) is calibrated implicitly to the *mean* of the observed distribution through the careful choice and selection of model parameters. It should be noted, however, that SPACSYS is not fitted or re-calibrated explicitly to external data.

Topological characteristics, such as the integrating effect of the catchment, could also contribute to this behavior. For example, large local slopes (that SPACSYS cannot represent) result in faster running water which, combined with intense rainfall, may result in higher peak flows that are not captured by SPACSYS. Over-predicted events are likely due to inaccurate representation of soil moisture, topography and other soil properties at the within-field scale, since SPACSYS simulates at the field scale (Liu et al., 2018). Despite these issues and the fact that our proposed hybrid approach was aimed at under-predicted extreme flow events, the hybrid approach resulted in more accurate forecasts and an increase in accuracy overall.

The CEM is usually used to describe the extreme dependence structure of the same variable at different sites or of different variables at the same site. In this study, we used the CEM in a bivariate context to model and link the same underlying state variable captured by different representational processes (i.e., direct measurement and PBM simulation of flow). The pseudo-observations obtained from the fitted model and based on the conditioning variable were aggregated to a single value which was then compared to the equivalent measured value. The same conditional simulations can be used to create confidence intervals that correspond to various scenarios and allow flexibility in choosing values according to the intended purpose.

In general, none of the applied criteria for the evaluation of the proposed hybrid method is sufficient singly; each of the model performance indices have strengths and weaknesses. The agreement indices are used mainly to investigate how accurately the model captures the dynamic of the temporal process. The error indices capture differences between the total flow or the volume of the hydrograph. Therefore, using both measures provides a more holistic evaluation of model performance. Since our main objective was to evaluate the performance of the proposed hybrid method in predicting extreme flows, the choice of the agreement indices is appropriate as they have been shown to be sensitive to peaks (Krause et al., 2005).

Despite the promising results obtained from the proposed methodology, it has the limitation of being tested for a specific case study site and for one PBM. Future research should, therefore, consider testing this approach for other catchment sites with different characteristics, as data-driven models need to be tested using a range of (large) datasets before applied in practice (Boulesteix et al., 2018; Papacharalampous et al., 2019; Tyralis et al., 2019). It would also be interesting to investigate whether and how the performance of SPACSYS, and by extension, the proposed techniques, would be affected by using forecasted weather variables as inputs instead of measured data to obtain the simulations. In real case scenarios, the threshold is defined commonly based on pre-existing information. Due to the nature of the NWFP experiment, it was not possible to define a threshold with physical meaning (e.g. likely flooding) with which to evaluate the estimated threshold. The threshold defines the peak flow events and consequently the training and testing datasets used in this research. Thus, it was not possible to define a threshold based strictly on the training dataset only as would normally be the case. However, we expect this to have a minimal effect on the results and not change the main conclusions drawn.

## Conclusions

In this research, we used a data-driven machine learning model (ELM) and a semi-parametric conditional model that stems from extreme value theory (CEM) to increase the accuracy of peak water flow events simulated by a PBM. The PBM most frequently under-predicted the maximum flows during a peak event, for which the CEM was applied, and over-predicted flows preceding and following it, for which the ELM was applied. The combined characteristics of the proposed methodology in general resulted in more accurate forecasts and improved representation of these peak events, according to several error and agreement indices. The detailed analysis undertaken in this research was developed based on simulated flow data obtained from only one PBM and for observed data at only one case study site. However, because of the general characteristics of the chosen PBM and of the proposed hybrid methodology, it is anticipated that the proposed approach will be suitable for a wide range of PBMs and water monitoring station schemes.

## Data Availability Statement

All North Wyke Farm Platform datasets (https://www.rothamsted.ac.uk/north-wyke-farm-platform) and the SPACSYS model (https://www.rothamsted.ac.uk/rothamsted-spacsys-model) are freely available. R software (R Core Team, 2019) was used for the implementation of the statistical models. The CEM was applied by using the texmex R package (Southworth et al., 2018), the elmNNRcpp R package was used for the ELM model (Mouselimis and Gosso, 2018) and the indices were calculated by using functions in the hydroGOF R package (Zambrano-Bigiarini, 2017).

## Author Contributions

SC: conceptualisation, methodology, software, formal analysis, writing–original draft, writing–review and editing. PA: conceptualisation, writing–review and editing, supervision, funding acquisition. AM: conceptualisation, writing–review and editing, supervision, funding acquisition. LW: software, writing–review and editing, supervision, funding acquisition. PH: conceptualisation, data curation, writing–review and editing, supervision, funding acquisition.

## Funding

Rothamsted Research receives grant aided support from the Biotechnology and Biological Sciences Research Council (BBSRC) of the United Kingdom. This research was funded by Rothamsted Research and Lancaster Environment Centre, the BBSRC Institute Strategic Programme (ISP) grant, “Soil to Nutrition” (S2N) grant numbers BBS/E/C/000I0320, BBS/E/C/000I0330 and the BBSRC National Capability grant for the North Wyke Farm Platform grant number BBS/E/C/000J0100.

## Conflict of Interest

The authors declare that the research was conducted in the absence of any commercial or financial relationships that could be construed as a potential conflict of interest.
